# Limited scientific coherence between global mental health research and indicators of science, health, mental health, and society: a longitudinal analysis across world regions

**DOI:** 10.3389/fpsyg.2025.1649735

**Published:** 2026-01-12

**Authors:** David A. Hernández-Paez, Mónica Acuña-Rodríguez, Nacira Maria Pertuz-López, Fabriccio J. Visconti-Lopez, Judith Cristina Martinez-Royert, Ivan David Lozada-Martinez

**Affiliations:** 1 Center for Meta-Research and Scientometrics in Biomedical Sciences, Barranquilla, Colombia; 2 Universidad de la Costa, Barranquilla, Colombia; 3 Universidad del Sinú, Cartagena, Colombia; 4 Universidad Científica Del Sur, Lima, Peru; 5 Facultad de Ciencias de la Salud, Centro de Investigaciones en Ciencias de la Vida, Universidad Simón Bolívar, Barranquilla, Colombia; 6 Biomedical Scientometrics and Evidence-Based Research Unit, Department of Health Sciences, Universidad de la Costa, Barranquilla, Colombia; 7 Clínica Iberoamérica, Barranquilla, Colombia

**Keywords:** evidence gaps, global health, mental health, mental health services, meta-research

## Abstract

**Introduction:**

Global mental health is a growing priority in health and development agendas. However, there is limited evidence on whether scientific research in mental health aligns with the health, societal, and developmental needs of different regions. The aim of this study was to analyze the associations between global mental health research and key indicators in science, health, mental health, and society across world regions.

**Methods:**

It was conducted a longitudinal analysis of 386,671 peer-reviewed publications on mental health (1886–2024) using data from five databases. We linked annual publication counts with 60 indicators across four domains (Economy, Development and Education; Global Health; Inequality and Poverty; and Governance and Rights). Associations were evaluated using linear regression models and standardized *β*₁ coefficients, stratified by World Health Organization regions.

**Results:**

Significant regional differences were observed in both publication volume and coherence with contextual indicators. Africa and South-East Asia, despite lower publication rates (<5%), showed strong associations between research output and gross domestic product per capita (*β* = 0.125, 95% CI: 0.05–0.19, *p* < 0.01), average years of schooling (*β* = 526.05, 95% CI: 30.5–1021.5), and physician density (*β* = 1,289.6, 95% CI: 322.2–2,257.1). Conversely, the Americas and Europe, which accounted for >75% of publications, showed weaker or non-significant associations with mental health burden indicators such as disability-adjusted life years and suicide mortality.

**Discussion:**

There is evidence of both alignment and misalignment between global mental health research and global needs. Scientific output does not consistently correspond to indicators of mental health burden or systemic needs.

## Introduction

Mental health is increasingly recognized as a foundational pillar of global health, human development, and sustainable societal progress (World Health Organization, n.d.; World Health Organization, n.d.). Defined by the World Health Organization (WHO) not merely as the absence of illness but as a state of well-being (World Health Organization, n.d.), mental health enables individuals to realize their potential, cope with life’s challenges, work productively, and contribute to their communities (World Health Organization, n.d.). As such, it is intimately linked to multiple domains of the 2030 Sustainable Development Goals, particularly those related to health, education, gender equality, economic growth, and the reduction of inequalities (Heymann and Sprague, 2023). In the post-pandemic era, the demand for effective mental health systems and research has gained unprecedented urgency, catalyzed by the widening global burden of mental disorders and their pervasive social and economic consequences (Chan et al., 2024).

Despite this recognition, global responses to mental health remain disjointed, inadequately funded, and frequently not aligned with the needs of the population (World Health Organization, n.d.). According to the WHO’s Comprehensive Mental Health Action Plan 2013–2030 (World Health Organization, n.d.), mental disorders are responsible for a significant proportion of years lived with disability worldwide, yet mental health care remains inaccessible for most (World Health Organization, n.d.). Mental health services are concentrated in urban settings and in high-income countries (HICs), while vulnerable populations in low- and middle-income countries (LMICs) face profound shortages in human resources, infrastructure, and governance (World Health Organization, n.d.; World Health Organization, n.d.). Furthermore, the 2022 World Mental Health Report reveals that ≤2% of national health budgets prioritize mental health, with the majority still funneled into institutionalized psychiatric care rather than community-based models recommended for universal health coverage and equity (World Health Organization, n.d.).

Beyond service provision, there is a critical knowledge and policy gap: global mental health research often does not reflect the epidemiological burden or social urgency of mental disorders across contexts (Zhou et al., 2018). As stated in both WHO reports, evidence-based interventions are essential, but equally important is ensuring that research priorities align with societal needs, are distributed equitably across regions, and inform action-oriented policies (World Health Organization, n.d.; World Health Organization, n.d.). However, few studies have systematically explored the alignment between global mental health research output and structural indicators of science, health, mental health, and societal development. This persistent disconnection between academic production and the goals of effective, context-sensitive mental health governance remains (Van Zyl et al., 2022).

To address this gap, we need a nuanced, regionally stratified understanding of the links between mental health research activity and broader developmental indicators. Such evidence is fundamental not only to assess scientific coherence but also to guide resource allocation, research funding, and policymaking that reflect actual needs across diverse global contexts (Tadesse et al., 2021). This study aims to analyze the scientific coherence between global mental health research and key indicators in science, health, mental health, and society using a cross-regional longitudinal approach. By quantifying and comparing these links across WHO regions, this study seeks to generate actionable insights into how research aligns, or misaligns, with population needs. The goal is to inform evidence-based strategies that foster coherence between research, policy, and investment, thereby supporting mental health systems strengthening, equity, and sustainable development worldwide (Gusenbauer and Haddaway, 2020; Lozada-Martinez et al., 2025b).

## Methods

### Study design

#### Longitudinal study

This study is classified under this methodological design because it incorporates repeated annual measures of research output and 60 contextual indicators across the same ecological units (countries and world regions) over multiple decades. In macro-epidemiology, scientometrics, and global health policy research, longitudinal designs do not require individual-level follow-up but rely on structured time-series or panel data that allow examination of temporal trends and dynamic associations (Lozada-Martinez et al., 2025b). Our dataset meets these criteria, as all variables retain consistent yearly observations for the same ecological units (Lozada-Martinez et al., 2025b).

### Data sources

To ensure methodological rigor and comprehensive coverage, a systematic search was carried out across a set of internationally recognized academic databases: Scopus, PubMed/MEDLINE, Web of Science Core Collection, SciELO Citation Index, and the KCI-Korean Journal Database. These sources were deliberately selected due to their wide geographical reach, the breadth of indexed content in health and biomedical sciences, and the high standards applied to peer-reviewed literature (Gusenbauer and Haddaway, 2020). By prioritizing databases with rigorous inclusion criteria and strong citation structures, the reliability and reproducibility of the analyses were reinforced. The use of these platforms is well-supported in previous studies of similar scope (Gusenbauer and Haddaway, 2020; Lozada-Martinez et al., 2025b; Andreasen et al., 2022), further strengthening the validity of our approach.

### Search strategy

To maximize precision and ensure reproducibility, a structured and conceptually grounded search strategy was developed based on Medical Subject Headings (MeSH) and their respective synonyms. The term ‘Mental Disorders’ (MeSH Unique ID: D001523) was used as the foundational descriptor. This third-order concept (Tree Number: F03) encompasses a recognized range of official mental disorders, allowing the inclusion of relevant synonyms. The aim was to systematically identify peer-reviewed publications that explored, analyzed, or evaluated mental health disorders. This approach enabled the retrieval of literature from a wide array of scientific disciplines, where the topic of mental health is relevant and actively studied.

In the initial phase, pilot searches were conducted to iteratively test combinations of descriptors and indexing tags across multiple databases and search engines. This allowed for the refinement and optimization of the search logic based on performance criteria such as relevance, sensitivity, and specificity. The final version of the strategy, which yielded the highest consistency and precision when tested in Scopus, was the following: TITLE-ABS-KEY (“Mental Disorder” OR “Mental Illness” OR “Mental Illnesses” OR “Psychiatric Disorders” OR “Psychiatric Disorder” OR “Psychiatric Diseases” OR “Psychiatric Disease” OR “Psychiatric Illness” OR “Psychiatric Illnesses” OR “Behavior Disorders” OR “Psychiatric Diagnosis”). This version was then adapted to match the structure and indexing frameworks of each additional platform used in the study, ensuring coherence and methodological comparability across sources (Supplementary material 1).

### Time period

The literature search was conducted on November 28th, 2024, in English, Portuguese and Spanish. The inclusion of these three languages responded to the need for capturing a broader and more representative spectrum of publications. The initial screening phase, focused on titles and abstracts, was carried out between November 30th and February 25th, 2025. This stage allowed for the preliminary identification of studies aligned with the objectives of this analysis.

### Eligibility criteria

To ensure the relevance and reliability of the data analyzed, studies were included in the analysis only if they met specific inclusion criteria. These were: (A) being peer-reviewed scientific publications indexed in regularly issued academic journals, which guarantees a minimum standard of editorial and scientific quality; (B) availability of the full-text version, which was essential to allow in-depth examination and data verification; and (C) a clearly defined general objective explicitly focused on the analysis, discussion, investigation, synthesis, or evaluation of any mental health disorder.

To preserve the integrity of the dataset, documents were excluded if they: (A) corresponded to publications with non-regular peer review process such as conference proceedings, books, book chapters, errata, or retracted articles; (B) lacked at least one essential bibliographic metadata (e.g., author names, journal title, or corresponding author information), which limited traceability and verification; or (C) were still in press at the time of the search and therefore lacked a finalized version suitable for analysis.

Although the primary focus was on English, Portuguese and Spanish literature, documents published in other languages were also eligible, provided they included an abstract in either English, Portuguese or Spanish and met all inclusion criteria while avoiding any exclusion criterion. Finally, considering the historical dimension of this study and its scope, no lower limit was applied to the year of publication, allowing for a longitudinal and comprehensive view of the evolution of the field.

### Data standardization

The results retrieved from all databases were exported in. CSV format, retaining the complete set of available metadata. This included essential elements such as document titles, author names and institutional affiliations, keywords, publication year, citation count, publication type, and other relevant descriptors. Preserving this information was fundamental to ensure the integrity of the selection and analysis process.

As an initial quality control step, four researchers independently performed a manual screening to eliminate duplicate records and to assess titles and abstracts according to the pre-established inclusion and exclusion criteria. This review was conducted using Microsoft Excel 2016, which allowed for structured visualization and systematic filtering of the retrieved data.

Following this, a second independent review phase was undertaken by the same four researchers to carry out detailed data extraction focused on scientometrics variables, indicators of science and society, and metrics related to global health and global mental health. In cases where discrepancies arose between reviewers in any of the two phases, an external evaluator was brought in to resolve disagreements through discussion and consensus.

To promote consistency across the dataset and strengthen comparability in subsequent analyses, key variables were standardized. For instance, all documents identified as reviews, regardless of their specific methodological format (narrative, systematic, or meta-analysis), were grouped under a unified category labeled “reviews.” Similarly, for the variable ‘country,’ the nationality of the corresponding author was used as the primary reference, ensuring a consistent geographic attribution across all records.

### Data synthesis and analysis

To assess the scientometrics characteristics of the included publications, data were extracted on two widely recognized journal-level metrics: the quartile ranking and the h-index, each adjusted to the publication’s corresponding reference year. These indicators were sourced from the historical archives of Scimago Journal & Country Rank (available from 1999 onward) and the Journal Citation Reports (available since 1997). For each journal, the highest available metric was selected to ensure consistency and capture the maximum potential visibility or impact associated with the publication venue. This approach allowed for a standardized evaluation of the scientific positioning of each article within the academic landscape over time.

To enable comparisons across contexts, countries were classified into six geographic regions according to the WHO’s regional groups: The Americas, Europe, Western Pacific, Eastern Mediterranean, South-East Asia, and Africa (World Health Organization, n.d.). This classification reflects widely accepted global health frameworks and facilitates alignment with global official reports. In addition, each country was categorized by income level based on the World Bank’s latest 2024 economic classification (World Bank, n.d.), which stratifies economies into four tiers: low-income (LICs), LMICs, upper-middle-income (UMICs), and HICs (World Bank, n.d.). Incorporating both geographic and economic stratifications provided a multidimensional lens for interpreting global publication trends and their associations with structural and contextual factors.

It was collected data on 60 indicators across all available countries and years from official international sources such as the WHO’s Global Health Observatory (World Health Organization, n.d.), the World Bank (World Bank, n.d.), United Nations World Population Prospects (United Nations, n.d.), and the Institute for Health Metrics and Evaluation (Institute for Health Metrics and Evaluation, n.d.). These indicators were grouped into four categories: (1) Economy, Development, and Education; (2) Health; (3) Inequality and Poverty; and (4) Governance and Rights. Data were retrieved via public APIs when available or downloaded manually. To enable comparability between regions for subsequent analyses, the indicators were summarized as shown in Supplementary material 2.

To examine the associations between the selected indicators and scientometrics variables, linear regression analyses were performed. Each indicator was evaluated in relation to the number of publications. Depending on the context of the specific analysis, variables were treated as either dependent or independent. To account for possible regional variation, models were developed separately for each WHO’s regional groups (World Health Organization, n.d.). Only statistically significant models with meaningful effect sizes are presented in the main results, while the full set of outcomes is included in Supplementary material 3. Additionally, we constructed a matrix of regression coefficients by converting scientometrics variables to standardized Z-scores.

To enhance transparency and reproducibility, all variables included in the regression analyses were explicitly operationalized according to their measurement level and source. The scientometrics variables (e.g., annual publication counts, h-index, quartile ranking) were treated as continuous or ordinal variables depending on their inherent scale, whereas country and region classifications were treated as nominal variables. All indicators retrieved from international databases (e.g., Gross Domestic Product [GDP] per capita, literacy rate, life expectancy, Disability Adjusted Life Years (DALYs), poverty rate, governance indicators) were used in their original continuous form, as reported by the respective institutions (WHO, World Bank, and others). No transformations were applied except for standardization (z-scoring) when producing the cross-regional comparative matrices.

In all models, publication volume was used as the dependent variable when assessing whether contextual indicators predicted research output. Conversely, indicators were used as dependent variables when the analytical objective was to examine how research activity related to specific health or societal outcomes. This bidirectional analytic structure reflects the ecological nature of the study, which aims to describe patterns of association rather than infer causation.

A meta-analysis was conducted on the regression results. For each health indicator, the regional regression coefficients and their corresponding standard errors served as input for a random-effects meta-analysis. This method incorporated regional heterogeneity and yielded pooled estimates of the associations. The between-region variance was estimated using the Restricted Maximum Likelihood (REML) method. Only significant findings from the meta-analyses are reported in the main text; comprehensive results are provided in Supplementary material 4.

All statistical analyses were conducted using R software (version 4.3.1). The scripts for these analyses, along with detailed annotations, are available at: https://doi.org/10.5281/zenodo.15161386.

### Ethical statements

This study was approved by the Scientific Committee of Universidad de la Costa (code GRA.2021-07-002-19). However, no humans, animals, or medical records were used as units of analysis.

## Results

### Global mental health research: regional trends and characteristics

**Figure 1:**
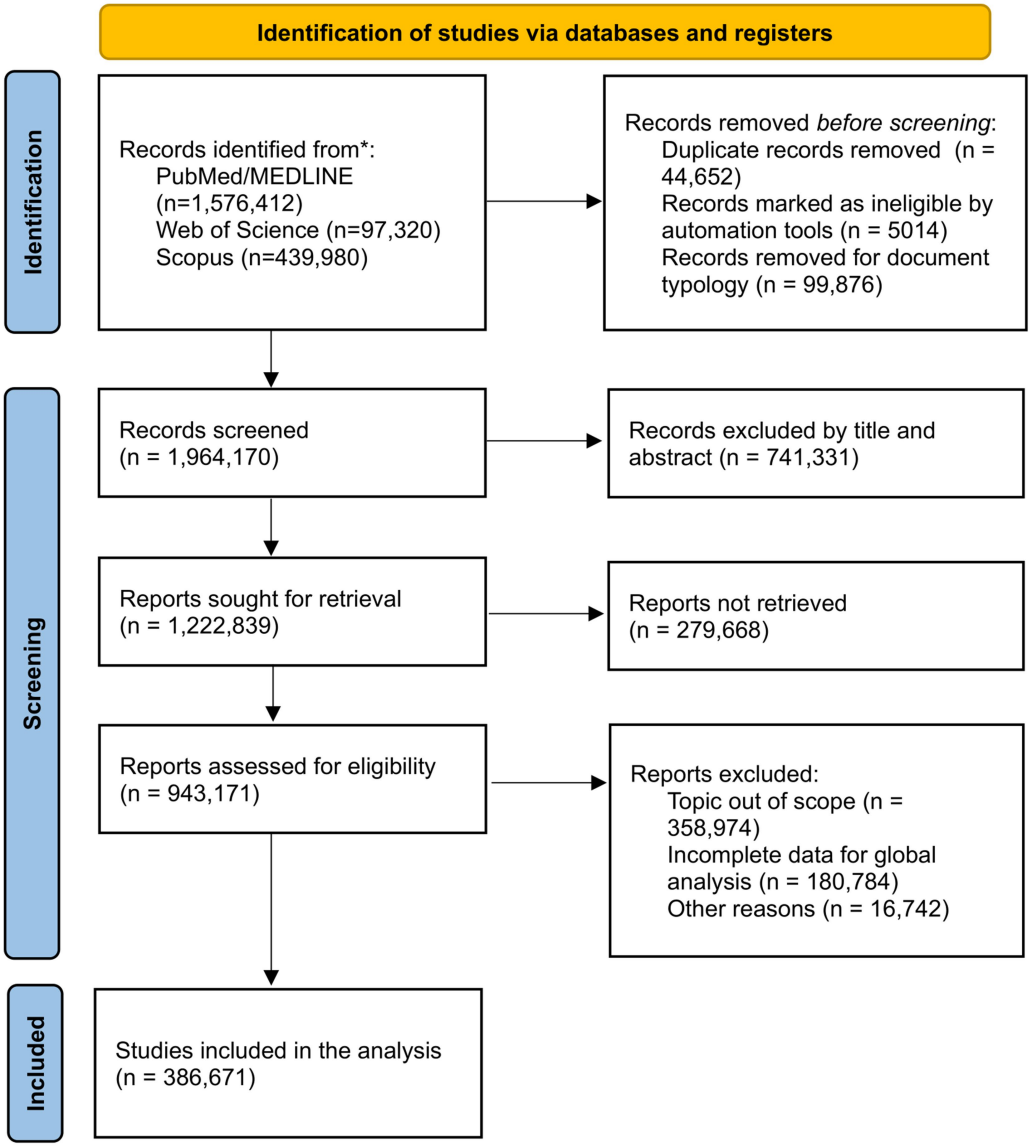
Flowchart of selected documents.

**Figure 2:**
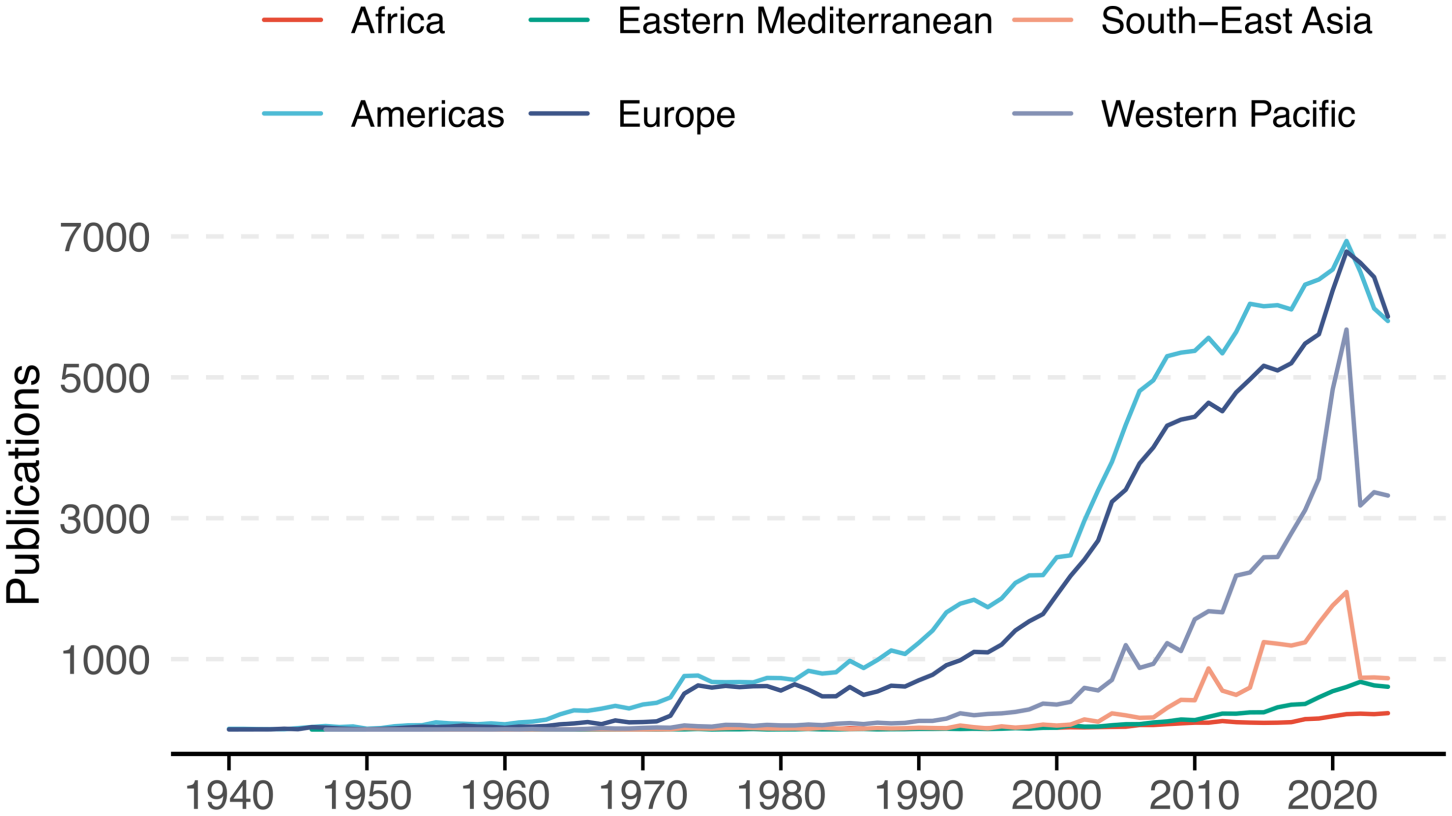
Annual trends in mental health-related publications by region (1940–2024). Variations in number of publications among the six regions over time.

A total of 386,671 documents were identified (Figure 1). The largest proportion of publications originated from the Americas (43%) and Europe (35.4%) (Table 1), with a relatively consistent number of publications across years (Figure 2). Although not shown, based on our search strategy, the earliest publication on mental health topics was from the Americas, published in JAMA in 1886 (Carpenter, 1886). The Eastern Mediterranean and African regions contributed the least, with 1.7 and 0.8% of total publications, respectively.

**Table 1:** General characteristics of articles on mental health by region (*N* = 386,671).

Magnitude	Americas	Europe	Western Pacific	South-East Asia	Eastern Mediterranean	Africa
Publications (%)	166,353 (43)	136,945 (35.4)	55,576 (14.3)	17,815 (4.6)	6,776 (1.7)	3,206 (0.8)
Total citations per paper, Mean	8,253,109 (49.6)	3,836,724 (28)	1,329,071 (23.9)	272,894 (15.3)	74,401 (10.9)	44,829 (13.9)
H-index, Mean (SD)	148.3 (126.7)	113.5 (96.3)	123 (92.4)	87.7 (75.4)	64.3 (66.2)	89.8 (87.9)
Gold/Bronze Open Access (ratio)	11,227/10,632 (1.05)	14,183/8,634 (1.6)	7,821/3,207 (2.4)	2,607/859 (3.03)	1,353/193 (7.01)	625/144 (4.3)
Document type (%)						
Article	128,886 (77.4)	106,587 (77.8)	45,526 (81.9)	14,180 (79.5)	5,993 (88.4)	2,737 (85.3)
Review	25,918 (15.5)	21,111 (15.4)	7,303 (13.1)	2,134 (11.9)	536 (7.9)	324 (10.1)
Letter	3,315 (1.9)	3,270 (2.3)	1,194 (2.1)	1,069 (6)	155 (2.2)	50 (1.5)
Editorial	3,290 (1.9)	2,317 (1.6)	585 (1.05)	162 (0.9)	41 (0.6)	40 (1.2)
Note	3,023 (1.8)	1732 (1.2)	596 (1.07)	173 (0.9)	24 (0.3)	42 (1.3)
Other	1921 (1.5)	1928 (1.7)	372 (0.78)	97 (0.8)	27 (0.6)	13 (0.6)
Journal quartile (%) (*n* = 329,796)						
Q1	96,537 (67.4)	61,601 (54.4)	28,581 (57.1)	5,706 (37.6)	1,458 (25.2)	1,120 (42.9)
Q2	31,142 (21.7)	23,138 (20.4)	12,214 (24.4)	4,119 (27.1)	1,331 (23)	574 (22)
Q3	11,289 (7.8)	15,365 (13.5)	5,637 (11.2)	3,458 (22.8)	1814 (31.4)	725 (27.8)
Q4	4,208 (2.9)	12,997 (11.4)	3,566 (7.1)	1869 (12.3)	1,161 (20.1)	186 (7.1)
Income group (%)						
High-income countries	157,518 (94.6)	128,271 (93.6)	27,516 (49.5)	0	896 (13.2)	2 (0.01)
Upper-middle-income countries	8,826 (5.3)	8,357 (6.1)	27,915 (50.3)	5,653 (31.7)	3,787 (55.8)	1,472 (45.9)
Lower-middle-income countries	9 (0.01)	317 (0.2)	145 (0.2)	12,162 (68.3)	2042 (30.2)	1,182 (36.8)
Low-income countries	0	0	0	0	51 (0.8)	550 (17.2)

The Americas contributed nearly half of all citations (49.6%) and included the most cited article, published in the Journal of Psychiatric Research, with over 77,000 citations (Folstein et al., 1975). The Eastern Mediterranean region had the fewest citations (10.9%) and the lowest average H-index (64.3). The highest average H-index values were observed in the Americas (148.3), Western Pacific (123), and Europe (113.5). The highest gold/bronze open access ratios were found in the Eastern Mediterranean (7.01) and South-East Asia (3.03), while the Americas (1.05) and Europe (1.6) had the lowest (Table 1).

Across all regions, original articles were the most common document type, particularly in the Eastern Mediterranean. Reviews were second, with the highest proportion in the Americas. Letters were less frequent, though South-East Asia had the highest share (6%). Of the total publications, 329,796 had a reported journal quartile. The Americas had the highest proportion of Q1 articles (67.4%), South-East Asia led in Q2 (27.1%), and the Eastern Mediterranean had the highest proportions of Q3 (31.4%) and Q4 (20.1%) publications. Q1 articles predominated in all regions except the Eastern Mediterranean.

**Figure 3:**
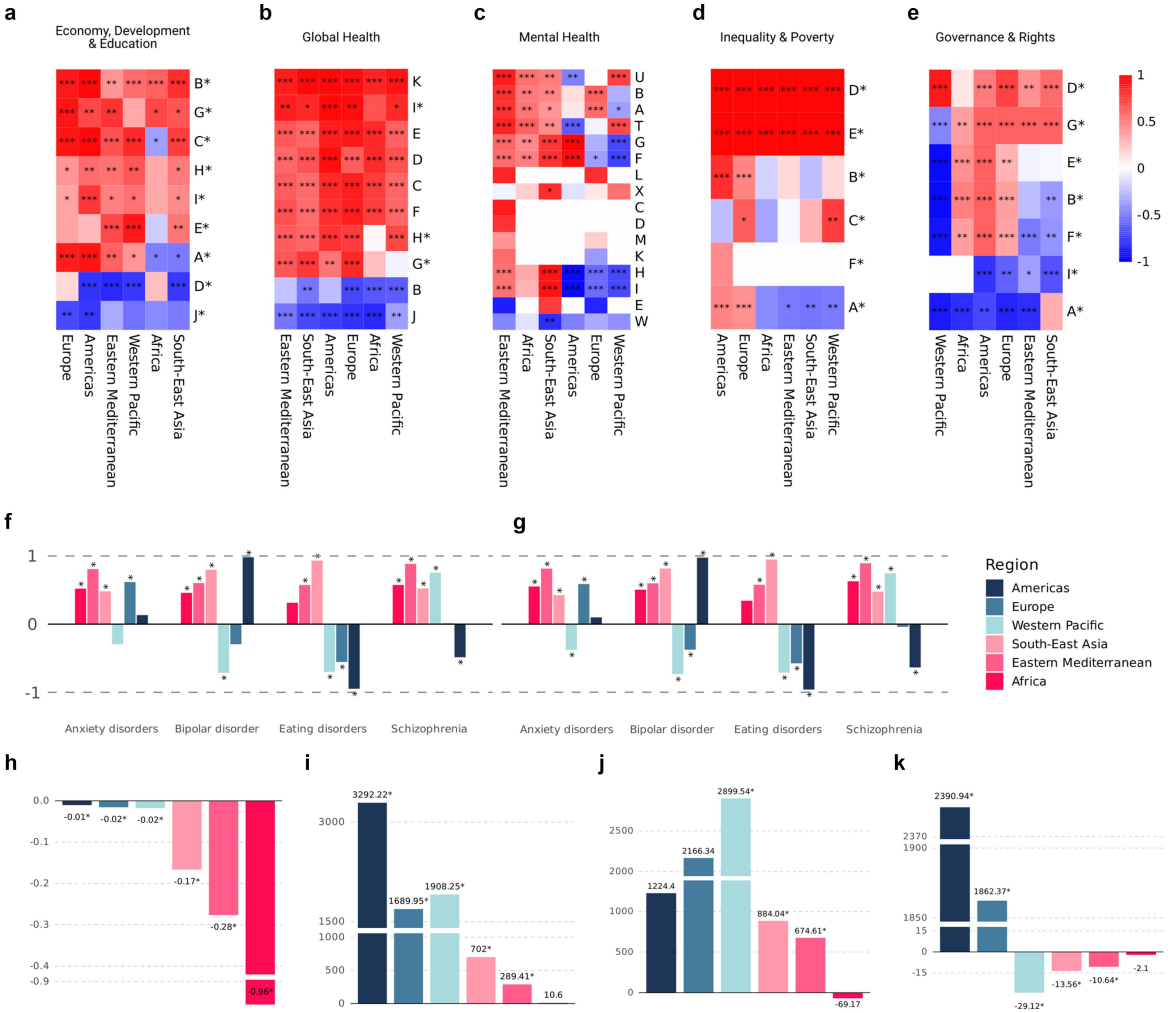
Normalized *β*₁ coefficients obtained via the *z*-score method across regions and groups of indicators (Supplementary material 2) **(a–e)**. *Z*-score β₁ coefficients for four mental disorders, assessed by prevalence **(f)** and disability-adjusted life years **(g)**, across WHO regions. Absolute β₁ coefficients for the following indicators modeled in relation to the number of publications, either as dependent or independent variables: child mortality rate **(h)**, number of physicians per 1,000 people **(i)**, research and development expenditure as a percentage of GDP **(j)**, and the proportion of the population living in extreme poverty **(k)** (Supplementary material 2). Asterisks (*) indicate variables used as independent variables in panels **(a–e)**, and denote statistical significance in panels **(f–k)**.

The correlations between the five domains of indicators and the different geographical regions are presented in Figures 3a–e.

### Lower mental health research output is associated with weaker impacts on mental health outcomes

Among the mental health disorders included, measured either by prevalence or by DALYs, we observed a tendency toward protective effects in regions with high publication rates (i.e., the Americas, Europe, and the Western Pacific), in contrast to regions with low publication rates (Figures 3f,g). Among these disorders, eating disorders showed the largest effect sizes, both in terms of prevalence and DALYs. Notably, this effect shifted markedly from a protective association in high-publication regions to a null or even contradictory positive association in low-publication regions, when using the number of mental health publications as the independent variable in the models. Specifically, the coefficients ranged from a 0.017-point decrease in the prevalence of eating disorders per additional mental health publication (SE = 0.0010, R^2^ = 0.899, *p* < 0.001) in the Americas, to a 0.048-point increase in prevalence (SE = 0.0266, R^2^ = 0.098; *p* = 0.081) in low-publication regions.

### Child mortality and research productivity show opposing regional patterns

Concerning global health indicators, we observed an inverse relationship between child mortality rates and regional publication rates, with regions exhibiting lower annual publication volumes showing the greatest reductions in child mortality (Figure 3h). Additionally, we used physician density (physicians per 1,000 people) as an independent variable to approximate the average scientific output per physician in each region. The Americas showed the highest association, with each additional physician per 1,000 people corresponding to approximately 3,292 more publications (SE = 323.07; R^2^ = 0.638; *p* < 0.001). Other regions also showed positive coefficients exceeding 1,000 publications, except for the Eastern Mediterranean and Africa, which showed 289 (SE = 60.88; R^2^ = 0.414; *p* < 0.001) and 10.6 (SE = 51.71; R^2^ = 0.001; *p* = 0.839), respectively, the latter being non-significant (Figure 3i).

### Global mental health publications and its economic and social correlates

Among economic indicators, Research and Development Expenditure (R&D) (% GDP) exhibited an inverse relationship in the group of regions with high publication rates. The highest coefficient was observed in the Western Pacific Region, where each additional percentage point of GDP allocated to research and development was associated with an increase of 2,899 publications (SE = 286.05; R^2^ = 0.811; p < 0.001). In contrast, the remaining regions showed positive but smaller coefficients, with the exception of the African Region, where a one-point increase in this indicator was associated with a decrease of 69.1 publications. However, this association was not statistically significant (SE = 85.77; R^2^ = 0.032; *p* = 0.429) (Figure 3j).

Interestingly, no observable effect was found for the share of the population living in extreme poverty on publication rates in the Americas and Europe, when using this indicator as the independent variable (Figure 3k). In all other regions, the coefficient was negative, indicating a reduction in publication volume with each percentage point increase in this poverty indicator.

### Mental health research and government indicators

As shown in Figure 3e, the majority of governance-related indicators used as independent variables exhibited positive coefficients. However, Indicators A and I [Functioning Government Index and Percentage of Territory Effectively Controlled by Government (Supplementary material 2)] showed predominantly negative and statistically significant coefficients. Similarly, the Western Pacific, Eastern Mediterranean, and South-East Asia regions displayed mostly negative coefficients across the governance indicators analyzed. In contrast, the Political Corruption Index showed positive and significant coefficients for nearly all regions, with the exception of Africa (Figure 3e).

### Meta-analysis of significant global indicators across regions

Following the meta-analysis of all regional regression coefficients, 11 indicators retained statistically significant effects (Table 2). Among the education-related indicators, both the average years of schooling and the adult literacy rate (% of individuals aged 15 and above) demonstrated positive and significant associations with publication rates. The average years of schooling yielded a coefficient of 526.05 (95% CI: 30.5–1,021.5), while the literacy rate showed a coefficient of 71.37 (95% CI: 1.73–141). Regarding economic indicators, R&D expenditure as a percentage of GDP and GDP per capita (current US$) emerged as positive stimulus for publication output.

**Table 2:** Significant meta-analysis results of indicators used as dependent or independent (*) variables in linear models related to number of publications.

Magnitude	Coefficient	(95% CI)	Variable	Q	τ^2^
Economy, development, and education indicators					
Average years of schooling	526.05	(30.5–1021.5)	ind	100.2*	308,127.6
Literacy rate, adult total (% of people ages 15 and above)	71.37	(1.73–141)	ind	42.5*	6,298.2
Research and development expenditure (% of GDP)	1,196.5	(252.7–2,140.3)	ind	116.6*	1,146,491
GDP per capita (current US$)	0.125	(0.05–0.19)	ind	186.6*	0.007
Global health indicators					
Life expectancy both sexes	213,848.4	(50,784.6 - 376,912.2)	dep	688.7*	4.04 × 10^1^⁰
Physicians (per 1,000 people)	1,289.6	(322.2–2,257.1)	ind	229.5*	1,425,333
Nurses and midwives (per 1,000 people)	472	(18.4–925.6)	ind	112.4*	306,834.6
Mental health indicators					
Self-reported life satisfaction	−0.0001	(0.0002–0.00003)	dep	18.05*	3.59 × 10^−9^
Inequality and poverty indicators					
Lifespan inequality: Gini coefficient in women	77.1	(55.13–99.08)	ind	5,955	753.2
Lifespan inequality: Gini coefficient in men	65.6	(48.5–82.6)	ind	3,989.6	453.4
Government indicators					
Functioning government index	−821.7	(−1624.7 - -18.7)	ind	114.8*	942,622

Among general health indicators, physician density exhibited a stronger association with publication rates compared to nurse and midwife density. Specifically, the coefficient for physician density was 1,289.6 (95% CI: 322.2–2,257.1), while that for nurse and midwife density was 472 (95% CI: 18.4–925.6). Additionally, when life expectancy for both sexes was used as a dependent variable, it showed a positive coefficient of 213,848.4 (95% CI: 50,784.6–376,912.2). Within mental health indicators, only self-reported life satisfaction retained a significant but small effect after the meta-analysis (Table 2).

Inequality and poverty indicators, measured through lifespan inequality via the Gini coefficient for both women and men, also maintained significant positive associations. The coefficients were 77.1 (95% CI: 55.13–99.08) for women and 65.6 (95% CI: 48.5–82.6) for men. Finally, among governance indicators, only the functioning government index remained significant after meta-analysis. It was negatively associated with publication output, showing a coefficient of −821.7 (95% CI: −1,624.7 to −18.7).

## Discussion

This study offers novel evidence to better understand the alignment between global mental health research and the broader needs of health systems, societies, and populations across different world regions. By integrating scientometrics data with indicators of science, health, mental health, and society, the study directly addresses a knowledge gap that has been repeatedly emphasized by WHO in both the Comprehensive Mental Health Action Plan 2013–2030 and the World Mental Health Report 2022: the lack of data to evaluate whether mental health research effectively responds to the most pressing needs in different global contexts (World Health Organization, n.d.; World Health Organization, n.d.). Prior to this analysis, assertions about the disconnect between research production and population-level needs were largely based on assumptions or descriptive data (World Health Organization, n.d.; World Health Organization, n.d.). This work now provides empirical evidence that allows for a deeper and more nuanced interpretation of that coherence, or lack thereof, by region, income level, and developmental indicator.

By generating a comparative and longitudinal overview across six WHO regions and four major dimensions, science, health, mental health burden, and societal development, this study helps clarify the degree to which research activity reflects structural challenges. For instance, our findings show that while the Americas and Europe account for the majority of mental health publications, their research output is not always proportionally aligned with indicators such as prevalence of mental disorders or gaps in service coverage. Conversely, regions like Africa and South-East Asia, where the burden of unmet mental health needs is substantial, exhibit low publication rates, but some strong associations between research activity and societal or developmental indicators. These findings reveal that mere volume of research is insufficient as a metric of impact and underscore the need for equity-sensitive indicators to evaluate scientific responsiveness. These patterns demonstrate long-standing asymmetries in global scientific investment and participation, but also suggest where future capacity-building and priority-setting can be better targeted (Authors, 2016).

The dominance of mental health research from HICs in the Americas and Europe can be attributed to structural advantages, including established academic institutions, robust funding, and ample human capital (Patelli et al., 2023). Despite these resources, research in these regions shows limited alignment with public health priorities, as evidenced by weak correlations between publication volumes and indicators such as suicide mortality and DALYs. This decoupling suggests a systemic misalignment between academic priorities and population health burdens, possibly driven by academic incentive structures that reward novelty and citation impact over local relevance. This finding reinforces the call for more balanced research agendas that not only produce knowledge but ensure its relevance to public health challenges (Yegros-Yegros et al., 2020).

In contrast, regions like Africa and the Eastern Mediterranean, despite contributing less to global mental health literature, demonstrated stronger statistical associations between publication counts and indicators like GDP per capita, literacy, and lifespan inequality. This suggests that even modest increases in research capacity in these regions may lead to proportionally higher relevance and responsiveness to real-world needs. Such findings highlight a critical opportunity: targeted investment in underrepresented regions may yield higher returns in societal alignment, supporting a more just global distribution of research benefits. For South-East Asia, the growing number of publications, particularly in recent years, alongside meaningful associations with health workforce and governance indicators, points to a potential positive shift in national research agendas toward local applicability (García Carrillo et al., 2024).

The positive correlations between research output and indicators such as average years of schooling, health workforce density, and R&D investment confirm the well-established link between knowledge ecosystems and development (Voda et al., 2023). However, this study goes further by illustrating how these associations vary by region and can help predict where strategic investments in science could translate into more targeted mental health responses. Our approach demonstrates that the maturity of a region’s research system is not only a function of investment, but also of its integrative capacity, how well science is embedded in governance, education, and social policy. The use of z-score standardization allowed us to normalize effect sizes and uncover nuanced patterns, such as the inverse associations in certain regions between research activity and extreme poverty, suggesting that inequality itself remains a barrier to both research participation and health equity (Malekzadeh et al., 2020).

Moreover, the associations between mental health publications and DALYs or life satisfaction indicators provide a window into the degree to which research is engaging with the most burdensome or socially significant mental health conditions. In regions where these associations are weak or absent, it may indicate that research is more academically driven or externally funded without strong local anchoring. This disconnect poses risks of epistemic injustice, where populations most affected by mental health burdens are least represented in research agendas, limiting both local policy utility and global knowledge diversity.

Before this analysis, global mental health research was often assessed in terms of volume and visibility, but rarely in terms of coherence with contextualized needs. This study marks a conceptual and empirical turning point: rather than treating research production as a goal in itself, it evaluates its alignment with indicators that matter to populations and policymakers (Zicker et al., 2015; Lozada-Martinez et al., 2025c; Lozada-Martinez et al., 2025a). The originality of this approach lies in its multidimensional integration of health, science, and social data, and in its potential to guide smarter, more ethical investments in mental health research globally (Zicker et al., 2015). In this sense, the findings contribute not only to academic knowledge but also to the broader goals of global health governance and equity (Acuña-Rodríguez et al., 2025a; Rátiva Hernández et al., 2023; Lozada-Martinez et al., 2022; Acuña-Rodríguez et al., 2025b).

This study has inherent limitations. First, the use of scientometrics data is influenced by database inclusion policies, language biases, and the visibility of journals from regions. To mitigate this, we included multiple databases (Scopus, Web of Science, PubMed, and others) and applied multilingual search strategies. Moreover, while the reliance on MeSH descriptors may not capture all relevant research, the controlled vocabulary ensures conceptual consistency and high sensitivity in topic identification. Second, some of the indicators, such as DALYs or governance indexes, are modeled estimates and not direct measurements. To address this, we employed robust meta-analytic techniques and sensitivity analyses across multiple models to ensure the stability of results. Furthermore, to account for heterogeneity, we stratified the analyses by WHO region allowing for more context-specific interpretations.

Given the multidimensional nature of the study and the integration of several global indicators across regions and time, the analytical approach inherently carries a risk of identifying associations that may not reflect causal relationships. To mitigate this concern, our analyses were designed exclusively to detect patterns of statistical association rather than infer causality. We therefore interpreted all coefficients within a correlational and ecological framework, avoided causal terminology, and emphasized that the observed associations may be influenced by unmeasured confounders, regional heterogeneity, or structural differences in data availability. This clarification reinforces that the study aims to characterize coherence between research output and contextual indicators, rather than establish causal pathways.

## Conclusion

This cross-regional longitudinal analysis provides the first comprehensive evidence on the associations between global mental health research and key indicators in science, health, mental health, and society. Overall, the findings confirm that significant associations do exist, though unevenly distributed across regions and indicator types, highlighting both coherence and gaps in how global research activity aligns with real-world needs.

Regions such as South-East Asia and Africa, despite contributing fewer publications, demonstrated stronger correlations between mental health research output and societal indicators like GDP per capita, literacy rate, and inequality in lifespan. In contrast, HIC regions like Europe and the Americas showed greater research volume but weaker associations with indicators of mental health burden, such as DALYs and suicide rates. This suggests that while scientific capacity is higher in some regions, its alignment with public mental health priorities remains suboptimal.

These findings are relevant for decision-makers. They offer a data-driven foundation to inform research funding, prioritize scientific agendas, and better allocate human and financial resources in line with health system demands. Understanding where scientific production is more responsive, or disconnected, from health and societal indicators enables global and regional stakeholders to foster more equitable, needs-based research ecosystems.

## Data Availability

The original contributions presented in the study are included in the article/Supplementary material, further inquiries can be directed to the corresponding authors.
