# Supplementary material for: Limited scientific coherence between global mental health research and indicators of science, health, mental health, and society: a longitudinal analysis across world regions
Source: Front Psychol. 2026 Jan 12;16:1649735. doi: 10.3389/fpsyg.2025.1649735 (PMC12833040; doi:10.3389/fpsyg.2025.1649735)
Supplement: Supplementary file 1 [file Supplementary_file_1.docx]

**Supplementary Material #1**

Search strategies used according to databases:

***Scopus database***

TITLE-ABS-KEY("Mental Disorder" OR "Mental Illness*" OR "Psychiatric Disorder*" OR "Psychiatric Disease*" OR "Psychiatric Illness*" OR "Behavior Disorders" OR "Psychiatric Diagnosis") = 438,980 documents found.

***PubMed database***

("Mental Disorders"[MeSH Terms] OR "Mental Disorder"[Title/Abstract] OR "Mental Illness*"[Title/Abstract] OR "Psychiatric Disorder*"[Title/Abstract] OR "Psychiatric Disease*"[Title/Abstract] OR "Psychiatric Illness*"[Title/Abstract] OR "Behavior Disorders"[Title/Abstract] OR "Psychiatric Diagnosis"[Title/Abstract]) = 1,576,412 documents found.

***Web of Science Core Collection, SciELO Citation Index, and the KCI-Korean Journal Database***

TS=("Mental Disorder" OR "Mental Illness*" OR "Psychiatric Disorder*" OR "Psychiatric Disease*" OR "Psychiatric Illness*" OR "Behavior Disorders" OR "Psychiatric Diagnosis") = 97,320 documents found.
