# Supplementary material for: Limited scientific coherence between global mental health research and indicators of science, health, mental health, and society: a longitudinal analysis across world regions
Source: Front Psychol. 2026 Jan 12;16:1649735. doi: 10.3389/fpsyg.2025.1649735 (PMC12833040; doi:10.3389/fpsyg.2025.1649735)
Supplement: Supplementary file 2 [file Supplementary_file_2.docx]

**Science, Global Health, Mental Health and Society Indicators Analysed**

| **Economy, Development, and Education indicators used for analyses (N= 10).** | | | |
| --- | --- | --- | --- |
| **Group** | **Summary Method** | **ID** | **Indicator** |
| Economy and Development (n =6) | Population-weighted average | A* | Current health expenditure (% of GDP) |
|  |  | B* | GDP per capita (current US$) |
|  |  | C* | Human Development Index |
|  |  | D* | Out-of-pocket expenditure (% of current health expenditure) |
|  |  | E* | Research and development expenditure (% of GDP) |
|  | Total sum across countries | F* | Charges for the use of intellectual property, payments (BoP, current US$) |
| Education (n = 4) | Population-weighted average | G* | Average years of schooling |
|  |  | H* | Literacy rate, adult total (% of people ages 15 and above) |
|  |  | I* | Literacy rate, youth total (% of people ages 15-24) |
|  | Weighted by the total population ages 15-64 | J* | Share of population with no formal education |
| ** Used as independant variables in the model.* | | | |

| **Health indicators used for analyses (N = 35).** | | | |
| --- | --- | --- | --- |
| **Group** | **Summary Method** | **ID** | **Indicator** |
| General heatlh (n = 11) | Population-weighted average | A | Disability-Adjusted Life Years (DALYs) |
|  |  | B | Homicide rate |
|  |  | C | Life expectancy at birth, total (years) |
|  |  | D | Life expectancy both sexes |
|  |  | E | Life expectancy of men |
|  |  | F | Life expectancy of women |
|  |  | G* | Nurses and midwives (per 1,000 people) |
|  |  | H* | Physicians (per 1,000 people) |
|  |  | I* | The Universal Health Coverage (UHC) Service Coverage Index |
|  | Weighted by the crude birth rate (per 1,000 people) | J | Child mortality rate |
|  | Total sum across countries | K | Deaths |
| Mental Health (n = 22) | Population-weighted average | A | Anxiety disorders (DALYs) |
|  |  | B | Anxiety disorders (Prevalence) |
|  |  | C | Beds for mental health in general hospitals (per 100,000) |
|  |  | D | Beds in community residential facilities (per 100,000) |
|  |  | E | Beds in mental hospitals (per 100,000) |
|  |  | F | Bipolar disorder (DALYs) |
|  |  | G | Bipolar disorder (Prevalence) |
|  |  | H | Eating disorders (DALYs) |
|  |  | I | Eating disorders (Prevalence) |
|  |  | J* | Government expenditures on mental health (% of health expenditures) |
|  |  | K | Mental health day treatment facilities (per 100,000) |
|  |  | L | Mental health outpatient facilities (per 100,000) |
|  |  | M | Mental health units in general hospitals (per 100,000) |
|  |  | N | Mental health units in general hospitals admissions (per 100,000) |
|  |  | O | Mental hospital admissions (per 100,000) |
|  |  | P* | Nurses in mental health sector (per 100,000) |
|  |  | Q | Outpatient visits (per 100,000) |
|  |  | R* | Psychiatrists in mental health sector (per 100,000) |
|  |  | S* | Psychologists in mental health sector (per 100,000) |
|  |  | T | Schizophrenia (DALYs) |
|  |  | U | Schizophrenia (Prevalence) |
|  |  | V | Social workers in mental health sector (per 100,000) |
| Subjective well-being (n = 2) | Population-weighted average | W | Self-reported life satisfaction |
|  | Average of the countries | X | Share of people who say they are happy |
| ** Used as independant variables in the model.* | | | |

| **Inequality and Poverty indicators used for analyses (N= 6).** | | | |
| --- | --- | --- | --- |
| **Group** | **Summary Method** | **ID** | **Indicator** |
| Inequality and Poverty | Population-weighted average | A* | Share of population living in extreme poverty |
|  | Average of the countries | B* | Gini index |
|  |  | C* | Income inequality: Atkinson index |
|  | Weighted by the Death rate, crude (per 1,000 people) | D* | Lifespan inequality: Gini coefficient in men |
|  |  | E* | Lifespan inequality: Gini coefficient in women |
|  | Weighted by the Multidimensional poverty headcount ratio (% of population) | F* | Multidimensional Poverty Index (MPI) |
| ** Used as independant variables in the model.* | | | |

| **Governance and rights indicators used for analyses (N= 9).** | | | |
| --- | --- | --- | --- |
| **Group** | **Summary Method** | **ID** | **Indicator** |
| Governance and Rights | Average of the countries | A* | Functioning government index |
|  |  | B* | Human rights index |
|  |  | C* | LGBT+ legal equality index |
|  |  | D* | Political corruption index |
|  |  | E* | Private civil liberties index |
|  |  | F* | Rigorous and impartial public administration index |
|  |  | G* | State capacity index |
|  | Population-weighted average | H* | Corruption Perception Index |
|  | Weighted by the total land area (km^2^) | I* | Percentage of territory effectively controlled by government |
| ** Used as independant variables in the model.* | | | |
